# Alaska Native genomic research: perspectives from Alaska Native leaders, federal staff, and biomedical researchers

**DOI:** 10.1038/s41436-020-0926-y

**Published:** 2020-08-25

**Authors:** Vanessa Y. Hiratsuka, Michael J. Hahn, R. Brian Woodbury, Sara Chandros Hull, David R. Wilson, Vence L. Bonham, Denise A. Dillard, The Alaska Native Genomics Research Workshop Group, Jaedon P. Avey, Andrea C. Beckel-Mitchener, Juliana Blome, Katrina Claw, Elizabeth D. Ferucci, Francine C. Gachupin, Armen Ghazarian, Lucia Hindorff, Sonya Jooma, Susan B. Trinidad, Jennifer Troyer, Hina Walajahi, Jaedon P. Avey, Andrea C. Beckel-Mitchener, Juliana Blome, Katrina Claw, Elizabeth D. Ferucci, Francine C. Gachupin, Armen Ghazarian, Lucia Hindorff, Sonya Jooma, Susan B. Trinidad, Jennifer Troyer, Hina Walajahi

**Affiliations:** 1 Southcentral Foundation, Anchorage, AK USA; 2 National Institutes of Health, Bethesda, MD USA; 3 Southcentral Foundation, Anchorage, AK USA; 4 National Institutes of Health, Bethesda, MD USA; 5 Department of Medicine-Bioinformatics, University of Colorado Denver, Aurora, CO USA; 6 Alaska Native Tribal Health Consortium, Anchorage, AK USA; 7 Department of Family and Community Medicine, University of Arizona, Tucson, AZ USA; 8 Department of Bioethics, University of Washington, Seattle, WA USA

**Keywords:** Alaska Natives, North American, ethics, US National Institutes of Health, social responsibility, trust

## Abstract

Meaningful engagement of Alaska Native (AN) tribes and tribal health organizations is essential in the conduct of socially responsible and ethical research. As genomics becomes increasingly important to advancements in medicine, there is a risk that populations not meaningfully included in genomic research will not benefit from the outcomes of that research. AN people have historically been underrepresented in biomedical research; AN underrepresentation in genomics research is compounded by mistrust based on past abuses, concerns about privacy and data ownership, and cultural considerations specific to this type of research. Working together, the National Human Genome Research Institute and two Alaska Native health organizations, Southcentral Foundation and the Alaska Native Health Board, cosponsored a workshop in July 2018 to engage key stakeholders in discussion, strengthen relationships, and facilitate partnership and consideration of participation of AN people in community-driven biomedical and genomic research. AN priorities related to translation of genomics research to health and health care, return of genomic results, design of research studies, and data sharing were discussed. This report summarizes the perspectives that emerged from the dialogue and offers considerations for effective and socially responsible genomic research partnerships with AN communities.

## INTRODUCTION

Meaningful engagement of Alaska Native (AN) tribes and the tribal health organizations (THOs) is essential to the conduct of socially responsible and ethical health research. Alaska Native people have historically been underrepresented in biomedical research, and this trend continues in modern genetic and genomic research.^1,2^

In 2018, two Alaska Native health organizations—Southcentral Foundation (SCF) and the Alaska Native Health Board (ANHB)—cosponsored a workshop on genomic research with the National Human Genome Research Institute (NHGRI). The goal of the workshop was to better understand barriers to and opportunities for engaging AN people in genomic research and explore the circumstances and policies that facilitate partnership and participation of AN peoples in community-driven genomics research. These three cosponsors each brought unique perspectives and stakeholders to the conversation. ANHB is a statewide advocacy organization serving over 230 tribes and associated THOs of Alaska, SCF is a regional THO serving over 65,000 American Indian (AI) and AN people, and the National Human Genome Research Institute (NHGRI) is a leading authority in the field of genomics research at the National Institutes of Health (NIH).

This meeting report describes themes and perspectives that emerged during the workshop discussions, including the health research priorities of AN people, the importance of community engagement, considerations for data sharing, and future directions. In describing the distinct opportunities, priorities, and challenges of biomedical, genomics, social science, and ethical, legal, and social implications (ELSI) research involving AN people, we hope to provide guidance for future research in this area.

## WORKSHOP PARTICIPATION AND DESIGN

Ninety-seven individuals representing three primary stakeholder groups attended the workshop: (1) AN tribal and village leadership and AN THOs, hereafter referred to as “tribal representatives” *N* = 49; (2) academic and biomedical researchers with substantial experience conducting research in collaboration with American Indian and Alaska Native (AIAN) peoples, hereafter referred to as “researchers” *N* = 29; and (3) federal agency representatives from the NIH and the Centers for Disease Control and Prevention Arctic Investigations Program, hereafter referred to as “federal agency representatives” *N* = 19 (see Supplementary Table S1 online). The majority of tribal representatives were associated with THOs as staff who work directly with researchers in clinical and tribal community settings or board members who regularly review research proposals in AN tribal and health settings. Researchers included individuals who were current and past partners in AN partnered clinical and biomedical research projects. The majority of federal agency representatives were associated with the NHGRI, with seven additional NIH institutes, centers, and offices represented.

The event was designed to encourage equal participation and dialogue between stakeholder groups. The agenda was organized into four sessions: priorities, opportunities, challenges, and data-sharing approaches for genomic research. Sessions began with panel presentations by representatives from each stakeholder group (see Supplementary Table S2) before transitioning to discussions among small groups of 8–10 attendees that were guided by a set of questions relevant to the session topic (see Table 1). The first panel and subsequent small group discussion aimed to explore some of the opportunities that genomic medicine may bring to bear on AN health. The second panel and associated small group discussion was aimed at exploring challenges and sought to identify and explore potential barriers and critical conversations that must be addressed before genomics research proceeds. Issues around genomics research such as study design and timing, return of results, and translation of findings into clinical settings were specific challenges that were considered. The final panel and small group discussion was aimed at exploring current examples of data sharing (e.g., biospecimen and associated data, networked biorepositories, de-identified data sets, secondary data analysis, data ownership, data destruction, and data use agreements) as well as concerns and considerations for AN communities, and proposing both policies and practices that can guide future research efforts.

**Table 1:** Introductory panel presentations.

Topic	Panelists	Small group discussion questions
**Opportunities panel** As noted in the NHGRI Strategic Plan, “Genomic discoveries will increasingly advance the science of medicine in the coming decades, as important advances are made in developing improved diagnostics, more effective therapeutic strategies, an evidence-based approach for demonstrating clinical efficacy, and better decision-making tools for patients and providers.” This session aims to explore some of the opportunities that genomic medicine may bring to bear on Alaska Native health.	**Gail P. Jarvik, MD, PhD** Head, Division of Medical Genetics The Arno G. Motulsky Endowed Chair in Medicine Professor of Genome Sciences University of Washington Medical Center **Lucia Hindorff, PhD** Program Director, National Human Genome Research Institute **Esai Twitchell Jr**. Board Member, Alaska Native Tribal Health Consortium	1. What are examples of how genomics discoveries can benefit patients, families, and communities today? 2. What are examples of the application of genomics for communities and health-care systems that can serve as models for Alaska Native communities and health-care systems? 3. How can tribal leaders, researchers, and officials effectively gather input from Alaska Native community members, health providers of care to Alaska Native people, and those in leadership positions within Alaska Native tribes and tribal organization on the opportunities most important to Alaska Native peoples’ health?
**Challenges panel** The potential for genomics research to improve health comes with several important considerations for individuals and communities. This session aims to explore some of those challenges, and to identify and explore potential barriers and critical conversations that must be addressed before genomics research proceeds. Issues around genomics research such as study design and timing, return of results, translation of findings into the clinic all present challenges that must be considered.	**Francine C. Gachupin, PhD, MPH** Associate Professor, University of Arizona **Jennifer Troyer, PhD** Program Director, National Human Genome Research Institute **Karen Caindec (Tlingit)** Secretary/Treasurer and Director, Southcentral Foundation	1. What are the top three obstacles to engagement with genomics research in Alaska Native communities? 2. What are the unique cultural considerations that researchers, partners, and funders should take into consideration when exploring genomics research with Alaska Native populations? 3. Are there lessons or best practices from other research studies or communities that can be applied to challenges faced in genomics research with Alaska Native people?
**Data-sharing approaches panel** Advances in research can occur when large numbers of people share their health information and associated biological samples. Not only is it common practice for scientists to share their data and samples with other scientists, it is an expectation of NIH funded research that generates genomic data. This session aims to explore current examples of data sharing, concerns and considerations for Alaska native communities, and propose both policies and practices that can guide future research efforts.	**Bert Boyer, PhD** Bob and Charlee Moore Endowed Professor Director, Alaska Native Health Research Department of Obstetrics and Gynecology Center for Developmental Health, Knight Cardiovascular Institute Oregon Health & Science University **Sara Chandros Hull, PhD** IRB and Bioethics Core Director, National Human Genome Research Institute **Sonya Jooma, MA** Policy Analyst, National Human Genome Research Institute **Charlene Naulty, MS**	1. What guidelines should govern data sharing for Alaska Native people? 2. What are some concerns or tensions related to current data-sharing models for Alaska Native communities relative to ideal models and possible options to address them? 3. What are some models of data sharing that are appropriate for Alaska Native communities?
**Priorities panel** Alaska Native tribes have a unique relationship with the US Government. Recognizing this, the NIH aims to foster respectful collaboration and informed decision-making in the research programs and opportunities in which Alaska Native people are involved. This session aims to discuss the areas of genomics research and kinds of programs focused on by tribal leaders and to identify research priorities that will advance health for Alaska Natives, taking into consideration the opportunities and challenges brought up in previous sessions.	**Wylie Burke, MD, PhD** Professor, University of Washington **Larry Brody, PhD** Director, Genomics and Society Division, National Human Genome Research Institute **Tina Woods, PhD (Aleut)** Senior Director, Community Health Services, Alaska Native Tribal Health Consortium	1. How can genomics research play an important part in the pursuit of better Alaska Native health? 2. What type of genomics projects could be pursued within Alaska Native tribes and health-care systems? 3. What priorities will require the most community engagement and endorsement?

*NHGRI* National Human Genome Research Institute, *NIH* National Institutes of Health.

Workshop organizers purposely arranged the composition of the discussion group to include representatives from each of stakeholder groups. Attendees were assigned to a different group for each session. A trained notetaker recorded the main points of discussion at each table, and a member from each discussion group summarized themes and highlights in a report out to the larger group. Sessions ended with open discussion among all attendees. The workshop concluded with a talking circle—an approach to sharing story that is common among AN cultures—in which all attendees were given an opportunity to contribute personal reflections and final thoughts on the event.

## COMMON THEMES WITH PREVIOUS AIAN RESEARCH

Challenges related to conducting research with AN communities resemble those seen in other minority communities. They include the mistrust of research, different research priorities, limited AN community research capacity, AN community and THO research burdens, institutional and logistical barriers, and misinformation and a lack of shared knowledge.^3–8^ Key points related to challenges in conducting research with AN communities were concerned with AN community-level review of research recognition and exploration of the unique backgrounds and health needs of individual AN tribes and groups, the community-level review processes in AN communities, and the multiple responsibilities of the tribal leaders involved in the community-level review processes.

Like many other Indigenous communities, AIAN people have experienced harm and ongoing stigmatization as a result of unethical and exploitative research practices.^9–11^ AIAN communities have also been misled about the purpose and promise of research or were involved in studies that expended local resources while failing to yield community benefits.^12^ Additionally, AN community members and tribal leaders had negative experiences with health-care services during the period when the Indian Health Service managed AN health care, and those experiences contribute to misgivings about the trustworthiness of federal and academic partners who say they will improve AN health.^4^ Negative experiences with social, environmental, and resource management research exacerbates skepticism among AN community members and tribes about the potential of genomic research and other forms of health research to benefit AN people through health-care improvements.

Each AN community has a distinct and culturally embedded set of norms, beliefs, and values that informs individual and community perspectives on research. Although AN people and the outside research community share many goals and ideals, differences between these groups exist. Among the differences discussed by workshop attendees, those related to research goals emerged most often. Scientists conduct research with the goal of building knowledge for its own sake but AN communities—whose participation in research expends limited resources and exposes them to potential harms—expect research to lead to tangible benefits for their communities.^10^ Prioritizing research as a means to improve AN health care limits the scope of research projects AN communities and THOs are willing to partner and devote resources to. This preference for applied research challenges genomic researchers who are unable to concretely demonstrate the immediate clinical utility of their proposals.

Limited research capacity within and across AN communities was one of the most commonly cited research challenges.^13,14^ Although AN communities include individuals with health-care backgrounds, many lack local experts trained in the science, technology, engineering, and math (STEM) disciplines, or who are experienced in law, project administration, grant writing, and ethics/research review—disciplines necessary in the research process. There is also limited or no access to the academic institutions and federal agencies that could partner with THOs and/or AN communities to develop local research capacity or directly provide these services and skillsets. Although a few THOs have developed robust health research departments staffed by AIAN researchers, the size of Alaska, the rurality of AN communities, and the health disparities experienced by AN people mean that additional culturally appropriate research capacity is warranted.

Tribal representatives also emphasized that researchers must recognize that participation in research imposes costs on communities and researchers need to adjust their requests accordingly to minimize burden and costs for the communities. Tribal leaders are responsible for all aspects of local government, including housing, safety, wildlife and resource management, sanitation, and health-care delivery. Time spent by tribal leaders engaging in a project, whether as a participant, coresearcher, or administrator, is time lost to other important activities. In communities that lack established research capacity and infrastructure, the burden imposed by research is exacerbated, since project-related activities are not part of anyone’s regular work. These burdens can be highest on AN Elders, who are often responsible for making key research-related decisions, including review of research proposals and dissemination products.

Attendees from all stakeholder groups also described institutional and logistical barriers to research. For example, grant timelines and funding restrictions set by research funders rarely account for or support the time-intensive processes of community engagement and relationship-building. In AN populations, where there is both a strong need to rebuild trust and a cultural emphasis on the value of relationships and communication, these formative activities are vital to the success of ethical, feasible, and community-driven research.

A lack of shared knowledge about one another’s worldviews—including culture, values, and conceptual frameworks—hinders the ability of researchers and community members to collaborate effectively on research projects and may lead to misunderstanding.^15^ AN community members may be unfamiliar with the technical language or methods of research or may be misinformed about the potential risks and benefits of genomic research. Similarly, researchers may lack knowledge of a community’s cultural norms, values, and beliefs; its perceptions and expectations of research; and its unique health needs.^10^ Since many harms caused by past research stemmed from an ignorance of AN community history, traditions, and protocols, addressing this lack of knowledge may be considered a moral imperative for researchers as a component of responsive justice.^16^

## UNIQUE OPPORTUNITIES AND CHALLENGES FOR RESEARCH WITH ALASKA NATIVE COMMUNITIES FROM WORKSHOP DIALOGUE

### Alaska Native health priorities

The leading causes of death for AN people are cancer, heart disease, and unintentional injury.^17^ AN leaders use health data to help guide advocacy, program planning, policy making, program evaluation, and research. SCF, as an example of one THO within the Alaska tribal health system, which is owned and operated by AN people, combines health-care delivery with research that addresses population-level health disparities. Tribal discussants emphasized that the focus of research within the Alaska tribal health-care system is the improvement of health and wellness in accordance with tribally determined health priority areas, and that research is permissible only when it directly aligns with and aims to produce translatable results for those health priorities. Tribal representatives described community benefit as the critical factor in weighing research risks and benefits. Tribal representatives reflected on past experiences in health care and with research that either lacked informed consent or lacked immediate personal and/or community benefit. In these cases, some community members felt like “guinea pigs”—research subjects used to test a new drug, product, or process without consent and with undue burden imposed on the individual and/or their community of which they are part. A key finding emphasized by tribal representatives was the need for fairness and equity in the distribution of the benefits and burdens proceeding from genomic research. This expectation is aligned with the bioethical principle of justice enumerated in the Belmont Report.^18^

The AN health-care system is chronically underfunded.^19,20^ Tribal representatives who lead THOs that deliver health care described need to compare the potential value of genomic research to the known value of other forms of health research, interventions, and health system improvements. The findings of genomic research may lead to expensive clinical tests that may not be covered by private or public insurance. Additionally, one tribal representative noted that any data collection integrated into the health-care system results in the use of resources by that system, and that even small changes in how data are collected can result in large downstream effects on resource use and availability. Researchers do not cover these unintentional costs; instead they must be absorbed by an already overtaxed health-care system. This makes it critical for communities with limited access to health-care resources to carefully weigh the costs and benefits of partnering in research. Discussants from all stakeholder groups noted that research funders should specifically support the building of research capacity and infrastructure in low-resource settings by providing additional resources to offset the burden of participation by the health system.

Some discussants thought genetic research should not be conducted unless applied clinical utility has been clearly established. Others, including tribal representatives, recognized that the potential benefits and applications of research—especially long-term benefits for future generations—cannot be fully known in advance. As expressed by one discussant, “We don’t always know going in if the research is going to benefit [the Alaska Native community]. How do you figure that out ahead of time to determine priorities?” Federal agency representatives and researchers attending the workshop thought that conducting ethical research required being transparent about this uncertainty and acknowledging that—given the long timelines characteristic of research—it can sometimes take decades to realize the benefits of research.

Federal agency representatives expressed that understanding the degree of heritability for some conditions could be potentially useful for the health-care decision-making of future generations. For example, community members may want to know whether a disease has a genetic etiology to understand “why” they or their children have a health issue. Individuals might want this information even in cases where no cure or intervention for the disease exists.^21^ Discussants thought that, through constructive dialogue, AN communities and researchers and federal agency representatives could determine whether to proceed with a study on the basis of values and benefits and risks to individuals and communities.^4^ One federal agency representative stated, “Research doesn’t guarantee a cure, but without it, there certainly won’t be a cure.”

Discussants noted that researchers should work with AN communities to identify genomic research questions and consider designs that are culturally appropriate and that maximize community health benefits. At the same time, researchers and federal agency representatives need to be aware of the broader needs and priorities of tribal communities and how these compete with research activities for finite individual and community resources. For example, attendees noted that the potential benefits of genomic research would likely assume low priority in communities without plumbing or reliable access to clean water. Attendees also advised conducting genomic research in the context of interventions and investigations that account for multiple determinants of health.^22^

### Existing infrastructure for AN-regulated research

Another unique aspect of biomedical and genomics research with AN people is the Alaska Area Specimen Bank (AASB), which today is a tribal–federal partnership controlled resource with guidelines and protocols pertaining to use of biological specimens. The AASB contains biospecimens dating back to 1961 that were collected from AN people, gathered for research and clinical testing conducted by the United States Public Health Service and Alaska Native THOs.^23^ In partnership with the federal government, AN people developed an oversight group staffed by tribal, state, and federal representatives that sets policies and provides procedures for banking specimens from research projects and for secondary research using banked specimens. Tribally driven research regulation in which AN people retain control over the management of biospecimens used in research is unprecedented. Many discussants noted that this model has the potential to be expanded or replicated in other communities. AN tribal representatives also stated that this approach to research governance made them more likely to approve of research projects that elected to store biospecimens in the AASB.

From a research perspective, AN health-care settings have several promising qualities. Most notably, researchers and federal agency representatives observed that many THOs have longitudinal electronic health record data on a large segment of the population and that this data could be paired with genetic data and other information to beneficial effect in health research. Federal agency representatives also stated that Alaska is home to many successful genetic research projects and that the multiple levels of tribal review (e.g., AN village review processes, regional/THO review processes, Indian Health Service Institutional Review Board, Alaska Native Tribal Health Consortium Health Research Review Committee) are defined and become navigable for researchers who are familiar with these processes. Several federal agency representatives and researchers compared these research review process with those employed by the Navajo Nation. Federal agency representatives were familiar with the Navajo Nation’s restriction on genetic research.^24^ However, they were less aware of AN review policies and processes for research review and data stewardship. AN policies and procedures for research review, specimen storage, and data stewardship are being considered as a model by AIAN tribes. Researchers who are working with AIAN tribes have been reviewing AN policies to update current processes on data-sharing and genetic research policies.^25^

### Community engagement in Alaska

Given the importance that AN culture places on relationships, researchers should be prepared to devote considerable time to community engagement efforts. One attendee observed that successful projects with AN communities are those in which the project staff and principal investigator(s) have spent extensive time developing relationships with the community. A tribal representative spoke about the difficulty of building trust and rapport with researchers from outside the community without opportunities for repeated and ongoing contact.

The logistics involved in conducting research in rural AN communities can pose significant challenges to researchers, as most rural communities are remote and cannot be accessed by road. In addition, inclement winter weather can make travel unpredictable, delayed, dangerous, or impossible. Communication infrastructures are frequently underdeveloped or unreliable and the distances between communities and the topography of Alaska are extreme by any standard. These factors inevitably increase the cost and time involved in transporting the materials, equipment, and personnel necessary for research, thereby creating resource restrictions that further constrain researchers’ ability to engage in the robust community engagement necessary for culturally appropriate research.^5^ Internet-based communications save time and money and attendees noted that they may help to sustain existing partnerships. However, they are dependent on reliable communications and technology infrastructure and are insufficient for developing relationships and trust in rural AN communities if not used in concert with in-person visits. Discussants described several examples of research, including genomic research, that were successful and conducted in a community-engaged manner, despite these challenges.^26,27^

Community demographics, conflicting schedules, and institutional change can also create challenges for study design.^3^ Results and recommendations from research conducted in Anchorage and in other urban hubs may not be generalizable or applicable to small rural communities throughout Alaska due to dramatic differences in the availability of health and social services and the extent of environmental factors that may be associated with certain health risks or that shape health-related behaviors. Researchers affiliated with academic institutions may try to conduct recruitment, data collection, and other time-intensive research activities during the summer, when their teaching obligations are reduced. However, this seasonal schedule conflicts with the traditional subsistence activities (e.g., moose hunting, whaling, salmon fishing) that are an important part of AN culture and occur during this same period. Attendees also noted that leadership changes in federal agency and tribal offices and in academic and community-based research institutions pose challenges to the development of sustained relationships and to trust in the permanence and enforceability of research agreements.

Attendees also pointed to the high value that academic/research institutions place on scientific journal publications compared with community engagement and dissemination efforts. The failure of appointment and tenure committees and NIH scientific review committees to recognize and reward researchers for their commitment to community engagement efforts was provided as evidence of perverse incentives that pressure researchers to maximize the speed and efficiency of research processes at the expense of community values. To some attendees, these policies suggest that, despite the use of participatory research approaches that prioritize engagement and collaboration, research institutions are beholden to and constrained by commitments and unable to effectively elevate community priorities over the interests of research institutions.

### Data sharing

Data sharing is one aspect of biomedical and genomic research that is often viewed with apprehension, especially among communities who have had negative experiences with research.^28^ Issues of privacy, stigmatization, and unintended consequences are frequently cited as reasons to restrict or prohibit data sharing.^4,13^ Attendees were asked to discuss issues related to data sharing and its impacts on AN people. The meeting attendees were not given a definition for “data sharing” to allow for an open-ended discussion of data types, data access, and data use. Data involved in the discussions included phenotypic and genotypic data, biological specimens, medical record data, and secondary data analysis. Discussions about data sharing indicated that AN leaders were not opposed to data sharing but stressed the importance of reciprocity, transparency, and respect. Themes included the risks versus benefits of data sharing, the process of consent and control of research data, and flexibilities that specific policies—such as the NIH Genomic Data Sharing Policy—have for AN communities.

Researchers must cultivate authentic relationships with AN communities to build trust. The dialogue that emerges from these relationships can guide how data should be shared, whether with restrictions or more openly. Communities will differ in what they desire for data sharing, and some may prefer active consent for every use.^28^ However, AN discussants emphasized a key finding that any respectful use of data would include first sharing findings with the communities involved in research prior to broader public dissemination. Some communities and THOs have codified this approach to results dissemination as a requirement for doing research, but acknowledgement and agreement on the part of researchers and federal agency representatives is important to building trust and collaboration.

More communication and education about the benefits and drawbacks of data sharing is critical to acceptance of these practices by AN communities. Within the framework of reciprocity, transparency, and respect, open dialogue is critical to the development of data-sharing guidelines, resources, and models that can be employed by AN communities and researchers. The issues identified by discussants included individual versus community consent, the secondary use of data, data security, and de-identification. Many discussants voiced concern about the potential for re-identification of shared data derived from a small population or linked to a specific geographic area, even in cases where shared data were aggregated or summary level.

AN communities rely on research review boards and community input to guide research and effectively communicate to participants and the community how research is reviewed, how data are used, and how to authorize future use of data.^18,29^ Review by institutional review boards as required by federal funders and academic institutions is oriented toward protecting individuals rather than communities. By contrast, community input throughout the research process helps ensure that important community health implications are considered and that researchers account for how the framing of findings could cause community harm or have other unintended consequences.

Discussants noted that data-sharing approaches must be tailored to the needs of individual AN communities. In areas with more robust community-level research review processes, data sharing is considered on a project-by-project basis. In regions without well-established research regulation capacity and infrastructure, there is need for better systems to determine how data sharing is handled and what institutions originating the research can or should do to support research review. In both cases, discussants emphasized that data-sharing decisions cannot be one-time decisions; ongoing conversations and interactions with communities are needed, especially when there is a change in tribal leadership.

In the context of federally funded research that generates genomic data, there is an expectation by federal sponsors that resulting data will be shared. However, federal agency representatives noted that the NIH Genomic Data Sharing Policy includes flexibilities and exceptions that can be tailored to address decisions made by AN communities about data sharing.^30^ The discussants identified the need for guidance about processes to obtain an exception to data submission and consequences if they make a request. The AN discussants stated more information and transparency regarding when exceptions have been historically granted by NIH, and process among stakeholders to pursue exception to data submission for prospective research with AN populations are needed. The complexity of the topic and variables involved made it clear that a way forward on data sharing will require iterative and ongoing consultation with AN communities that cannot be achieved in a short workshop.

## NEXT STEPS, OPPORTUNITIES, AND RECOMMENDATIONS

### Strengths and limitations of the workshop

There was a consensus among attendees that the format of the workshop led to better dialogue between stakeholder groups and contributed to improved understanding of the issues. However, the organizers acknowledged that not all areas of Alaska were represented and underscored a need for continued engagement with more remote communities, villages with fewer resources, and AN groups not currently participating in any kind of research.

### Summary

As AN tribes, THOs, and federal partners continue to pursue opportunities to engage AN people in research, the stories shared at the Alaska Native Genomic Workshop highlighted THO research review processes, issues for discussion in the development of data-sharing agreements with federal partners, and areas to explore for additional grant funding to support AN health priorities. AN participation in genomics and other biomedical research is predicated on effectively incorporating AN community-level review. The uniqueness of the AN health-care system provides an opportunity to meaningfully combine delivery of care with research that furthers community health priorities. The focus of research within the AN health-care system is the improvement of health and wellness within THO determined health priority areas and any associated research should directly align with and aim to produce translatable results for those health priorities. Given the importance that AN culture places on relationships, researchers should be prepared to devote considerable time to community engagement efforts. In AN populations, where there is both a strong need to rebuild trust and a cultural emphasis on the value of relationships and communication, these activities are vital to the success of ethical, community-based research. Research and data-sharing decisions cannot be one-time decisions; there need to be ongoing conversations and interactions with communities, especially as tribal leadership is always changing. Participants voiced a great responsibility to continue these conversations and build upon the shared understanding and engagement begun in this meeting.

### Moving forward

Many discussants identified additional steps that stakeholders must take to identify specific genomic research priorities among AN communities. Many identified gaps in knowledge among community members, providers, and leaders. These gaps can be filled by providing more information to community members about what research is, how to identify high-quality versus low-quality science, and how to determine whether and for whom research may be beneficial or harmful. Building general awareness about research and familiarity with genetic research will require additional engagement and outreach. Researchers and federal agency representatives indicated that health-care providers who are familiar with genetic testing capacity and who keep up with advances in genomic medicine are well positioned to function as health communicators on genetics.

Some researchers and tribal leader discussants were interested in a fundamental shift in research in which community needs are identified first, followed by an exploration of whether research could even play a role in addressing these priorities. If research could potentially answer or address important priorities, this shift would empower tribes and THOs to issue calls for research to which scientists could respond. Discussants called for research to develop models in which control over research questions was located in participating communities.

Attendees held a shared perspective on how to build and strengthen partnerships to address the gaps in research capacity, including the administration of grants and related activities required for successful implementation. They strongly emphasized the need to cultivate interest in STEM disciplines and careers among AN youth and to provide AN students with the educational opportunities and financial resources necessary to achieve success in the competitive fields of health-related research in general and genetic research in particular. The benefits for youth and the greater community of increasing the representation of AIAN people in the health-care and health research workforce have been previously reported.^31^ Attendees cited several current programs ranging from Alaska’s long-running community health aide program to NIH funding for Native American Research Centers for Health but also emphasized the need for more programs tailored to the unique needs and challenges of AN scholars, many of whom experience a tension between community and scientific priorities that can force them to choose between professional success and allegiance to their communities. Attendees also spoke of the need to extend the research strengths already present in some THOs to other organizations and communities across Alaska.

Tribal attendees repeatedly stated that this event was an example of the kind of engagement with researchers that most AN communities are seeking, insofar as it brought together multiple AIAN communities, researchers experienced in partnering with these communities, and federal agency representatives to discuss the complexities of research approach and design. Many attendees commented that a sense of collegiality and personal commitment to community engagement developed over the course of the workshop. Discussants from all stakeholder groups appreciated that the workshop format allowed for an uncommonly candid and open discussion. The inclusion of both youth and Elder viewpoints as tribal leaders was felt to be particularly important. However, this kind of engagement is rare and expensive and requires the commitment of leadership and community partners to successfully rebuild trust and engagement with AN people.

In the past, ongoing dialogue between the tribal and scientific community has led to the successful development of data-sharing policies, as shown with the AASB policy and procedures.^23^ In the time since the Alaska Native Genomic Research Workshop, NIH facilitated a data-sharing and use agreement between the Navajo Nation and grantees of the NIH Environmental influences on Child Health Outcomes (ECHO) Program, a nationwide research consortium with a large-scale database. The agreement creates specific provisions related to tribal data protections, sharing data with ECHO consortium members, and interpreting results for publications. Such data-sharing agreements are rare, but with ongoing dialogue between federal funders, research scientists, tribal leaders, and Alaska Native community members, the ongoing development of tribally driven research and mutually beneficial agreements can set a new course in genomics and biomedical research.

## Supplementary information

Supplementary Tables
