# Supplementary material for: Alaska Native genomic research: perspectives from Alaska Native leaders, federal staff, and biomedical researchers
Source: Genet Med. 2020 Aug 25;22(12):1935–43. doi: 10.1038/s41436-020-0926-y (PMC7708301; doi:10.1038/s41436-020-0926-y)
Supplement: Supplementary file 1 — Supplementary Tables [file 41436_2020_926_MOESM1_ESM.pdf]

Supplementary Table 1: Alaska Native Genomic Research Workshop, Participant Roster

First Name	Last Name	Degree(s)	Organization Affiliation
Charles	Anderson		Southcentral Foundation
Charlene	Apok	MA	Southcentral Foundation
Michelle	Aregood		Southcentral Foundation
Bernadine	Atchison		Kenaitze Indian Tribe
Jaedon	Avey	PhD	Southcentral Foundation
Brian	Barnes	PhD, BS	University of Alaska Fairbanks
Andrea	Beckel-Mitchener	PhD	National Institute of Mental Health
Juliana	Blome	PhD	Environmental influences of Child Health
Verne	Boerner		Outcomes Alaska Native Health Board
Clara	Boerner		Alaska Native Health Board
Vence	Bonham	JD	National Human Genome Research Institute
Bert	Boyer	PhD	Oregon Health Sciences University
Larry	Brody	PhD	National Human Genome Research Institute
Tamara	Brown		Southcentral Foundation
Wylie	Burke	PhD, MD	University of Washington
Karen	Caindec		Southcentral Foundation
Esther	Cetina	MS	Southcentral Foundation
Karen	Cheung	MPH	Southcentral Foundation
Ashley	Christiansen	MBA	Southcentral Foundation
Katrina	Claw	PhD	University of Washington
Noah	Collins		Alaska Native Tribal Health Consortium
Louie	Commack		Maniilaq Association
Verlyn	Corbett	MD	Southcentral Foundation
Denise	Dillard	PhD	Southcentral Foundation
Carla	Easter	PhD	National Human Genome Research Institute
Doug	Eby	MD, MPH	Southcentral Foundation
Michael	Engelgau	MD, MS	National Heart, Lung, and Blood Institute
David	Farve		Southcentral Foundation
Elizabeth	Ferucci	MD	Alaska Native Tribal Health Consortium
Joshua	Franks		Cook Inlet Tribal Council, Inc.
Francine	Gachupin	PhD, MPH, CIP	University of Arizona
Donna	Galbreath	MD	Southcentral Foundation
Bart	Garber		Kenaitze Indian Tribe
Stephen	Gerrish		Tanana Chiefs
Armen	Ghazarian	PhD	National Cancer Institute
Jasmine	Gil		Alaska Native Tribal Health Consortium
Greta	Goto		Bristol Bay Native Corportation
Kevin	Gottlieb	DDS	Southcentral Foundation
Katherine	Gottlieb	MBA, DPS	Southcentral Foundation
Tiffany	Guinn	MA	Southcentral Foundation

Michael	Hahn		National Human Genome Research Institute
Tom	Hennessy	MD, MPH	Centers for Disease Control and Prevention
Lucia	Hindorff	PhD	National Human Genome Research Institute
Vanessa	Hiratsuka	PhD, MPH	Southcentral Foundation
Matt	Hirschfield	MD	Southcentral Foundation
Mike	Hirst		Southcentral Foundation
Sara	Hull	PhD	National Human Genome Research Institute
Roy	Hundorff		Southcentral Foundation
Gail	Jarvik	PhD, MD	University of Washington
Andrew	Jimmie		Tanana Chiefs Conference
Sonya	Jooma	MA	National Human Genome Research Institute
David	Koeller	MD	Oregon Health Sciences University
Cassandra	Kroto		Native Village of Tyonek
April	Kyle		Southcentral Foundation
Breanna	Lameman		Alaska Native Tribal Health Consortium
Ted	Mala	MD	Southcentral Foundation
Spero	Manson	PhD	University of Colorado Denver
Marjorie	Mau	MD, MS, MACP	University of Hawaiʻi at Mānoa
Brian	McMahon	MD	Alaska Native Tribal Health Consortium
Denise	Morris		Southcentral Foundation
Frederick	Murray		Norton Sound Health Corporation
Sarah	Nash	PhD, BA, MPH	Alaska Native Tribal Health Consortium
Charlene	Naulty		Aleut community of St. Paul Island
Jaylene	Person-Nyren		Southcentral Foundation
Terry	Powell	BBA	Alaska Native Tribal Health Consortium
Ricco	Ramos		Southcentral Foundation
Dara	Richardson-Heron	MD	All of Us Research Program
Austen	Rogers		Southcentral Foundation
Mike	Ryan-Todd	MA	Southcentral Foundation
Oliver	Sam		Southcentral Foundation
Krista	Schaefer	MPH	Southcentral Foundation
James	Segura		Southcentral Foundation
Aliassa	Shane		Southcentral Foundation
Jennifer	Shaw	PhD	Southcentral Foundation
Terry	Simpson	MD	Southcentral Foundation
Rosalyn	Singleton	MD, MPH	Centers for Disease Control and Prevention
Marie	Stewman	MBA	Southcentral Foundation
Ileen	Sylvester	MBA	Southcentral Foundation
Larissa	Tapia		Southcentral Foundation
Timothy	Thomas	MD	Alaska Native Tribal Health Consortium
Shirley	Thompson		Southcentral Foundation

Steve	Tierney	MD	Southcentral Foundation
Michelle	Tierney	PhD	Southcentral Foundation
Susan	Trinidad	MA	University of Washington
Jennifer	Troyer	PhD	National Human Genome Research Institute
Alberta	Unok		Alaska Native Health Board
Malia	Villegas	EdD	Native Village of Afognak
Hina	Walajahi		National Human Genome Research Institute
June	Walunga		Norton Sound Health Corporation
Kyle	Wark	MA	Southcentral Foundation
Laurie	Wiese		Southcentral Foundation
Scott	Williams		Southcentral Foundation
David	Wilson	PhD	National Institutes of Health, Tribal Health Research Office
Abbie	Wolfe	MA	Alaska Native Tribal Health Consortium
R. Brian	Woodbury		Southcentral Foundation
Tina	Woods	PhD	Alaska Native Tribal Health Consortium
Diana	Zirul		Kenaitze Indian Tribe

Supplementary Table 2: Alaska Native Genomic Research Workshop - Agenda
Location: Nuka Learning and Wellness Center, Tribal Drum room, 4085 Tudor Centre Drive, Anchorage, Alaska 99508

Day 1<br>July 10, 2018	8:00 – 8:15 am	Blessing<br>Ileen Sylvester
	8:15 – 8:30 am	Welcome<br>Chief Andrew Jimmie (Alaska Native Health Board [ANHB] Chair) and Verné Boerner (ANHB CEO)<br>Katherine Gottlieb (Southcentral Foundation [SCF] President/CEO)<br>Vence Bonham (National Human Genome Research Institute[NHGRI])
	8:30 – 9:00 am	Introductions<br>Moderator: Charlene Aqpik Apok, SCF
	9:00 – 9:15 am	Charge for the Workshop<br>Denise Dillard (SCF) and Vence Bonham (NHGRI)<br>· Acknowledgement of the work done to date<br>· Opportunities, priorities, and challenges in moving forward to expand genetic research and genomic medicine
	9:15 – 9:45 am	National Institutes of Health (NIH) Overview<br>Dave Wilson<br>· Describe the mission and vision for the NIH Tribal Health Research Office<br>· Discuss engagement of tribal communities with NIH
	9:45– 10:15 am	NHGRI Overview<br>Larry Brody, Lucia Hindorff, Jennifer Troyer<br>· Mission and vision and structure<br>· Research priorities<br>· Integration of genomics into clinical care<br>· Ethical, legal, and social implications of genomics research
	10:15 – 11:30 am	NIH Research Overview<br>Juliana Blome (Environmental influences on Child Health Outcomes [ECHO] Program)<br>Andrea Beckel-Mitchener (National Institute of Mental Health)<br>Michael Engelgau (National Heart, Lung, and Blood Institute)<br>Armen Ghazarian (National Cancer Institute)<br>Dara Richardson-Heron (*All of Us* Research Program)
	11:30 – 12:30 pm	Lunch
	12:30 am – 1:00 pm	Genomic Research Opportunities Panel<br>Tribal Leader: Esai Twitchell Jr.<br>Researcher: Gail Jarvik<br>NIH: Lucia Hindorff

	1:00 – 1:45 pm	Small Group Discussion on Opportunities
	1:45 – 2:30 pm	Report out on Opportunities
	2:30 – 2:45 pm	Break
	2:45 – 3:15 pm	Challenges Panel (Tribal leader, researcher, NIH)<br>Tribal Leader: Karen Caindec<br>Researcher: Francine Gachupin<br>NIH: Jennifer Troyer
	3:15 – 4:00 pm	Small Group Discussion on Challenges
	4:00 – 4:30 pm	Report Out on Challenges
	4:30 – 4:45 pm	Participant Reflections
	4:45 – 5:00 pm	Closing and Next Day Preview
	5:00 – 6:00 pm	Networking Reception
Day 2<br>July 11, 2018	8:00 – 8:15 am	Blessing<br>Lisa Dolchok (SCF Tribal Doctor)
	8:15 – 8:30 am	Welcome Back<br>Chief Andrew Jimmie (ANHB Chair) & Verné Boerner (ANHB CEO), Katherine Gottlieb (SCF President/CEO), Vence Bonham (NHGRI)
	8:30 – 9:00 am	Other Participant Reflections from Day One<br>Moderator: Charlene Aqpik Apok, SCF<br>Charge for Day Two<br>Denise Dillard and Vence Bonham
	9:00 – 9:45 am	Data Sharing Approaches Panel (Tribal leader, researcher, NIH)<br>Tribal Leader: Charlene Naulty<br>Researcher: Bert Boyer<br>NIH: Sara Hull, Sonya Jooma
	9:45 – 10:30 am	Small Group Discussion on Data Sharing Approaches
	10:30 – 11:30 am	Report Out on Data Sharing Approaches
	11:30 – 12:30 pm	Lunch
	12:30 – 1:00 pm	Priorities Panel (Tribal leader, researcher, NIH)<br>Tribal Leader: Tina Woods<br>Researcher: Wylie Burke<br>NIH: Larry Brody
	1:00 – 1:45 pm	Small Group Discussion on Priorities
	1:45 – 2:30 pm	Report out on Priorities
	2:30 – 3:30 pm	Participant Reflections
	3:30 – 4:00 pm	Workshop Closing<br>Denise Dillard and Vence Bonham
